# The celiac ganglion modulates LH-induced inhibition of androstenedione release in late pregnant rat ovaries

**DOI:** 10.1186/1477-7827-4-66

**Published:** 2006-12-21

**Authors:** Marilina Casais, Silvia M Delgado, Zulema Sosa, Carlos M Telleria, Ana M Rastrilla

**Affiliations:** 1 Laboratorio de Biología de la Reproducción (LABIR), Facultad de Química, Bioquímica y Farmacia, Universidad Nacional de San Luis. San Luis 5700, Argentina; 2 Division of Basic Biomedical Sciences, Sanford School of Medicine of the University of South Dakota, Vermillion, South Dakota 57069, USA

## Abstract

**Background:**

Although the control of ovarian production of steroid hormones is mainly of endocrine nature, there is increasing evidence that the nervous system also influences ovarian steroidogenic output. The purpose of this work was to study whether the celiac ganglion modulates, via the superior ovarian nerve, the anti-steroidogenic effect of LH in the rat ovary. Using mid- and late-pregnant rats, we set up to study: 1) the influence of the noradrenergic stimulation of the celiac ganglion on the ovarian production of the luteotropic hormone androstenedione; 2) the modulatory effect of noradrenaline at the celiac ganglion on the anti-steroidogenic effect of LH in the ovary; and 3) the involvement of catecholaminergic neurotransmitters released in the ovary upon the combination of noradrenergic stimulation of the celiac ganglion and LH treatment of the ovary.

**Methods:**

The ex vivo celiac ganglion-superior ovarian nerve-ovary integrated system was used. This model allows studying in vitro how direct neural connections from the celiac ganglion regulate ovarian steroidogenic output. The system was incubated in buffer solution with the ganglion and the ovary located in different compartments and linked by the superior ovarian nerve. Three experiments were designed with the addition of: 1) noradrenaline in the ganglion compartment; 2) LH in the ovarian compartment; and 3) noradrenaline and LH in the ganglion and ovarian compartments, respectively. Rats of 15, 19, 20 and 21 days of pregnancy were used, and, as an end point, the concentration of the luteotropic hormone androstenedione was measured in the ovarian compartment by RIA at various times of incubation. For some of the experimental paradigms the concentration of various catecholamines (dihydroxyphenylalanine, dopamine, noradrenaline and adrenaline) was also measured in the ovarian compartment by HPLC.

**Results:**

The most relevant result concerning the action of noradrenaline in the celiac ganglion was found on day 21 of pregnancy resulting in the inhibition of androstenedione release from the ovarian compartment. In addition on day 15 of pregnancy, LH placed in the ovarian compartment led to an inhibition of the release of androstenedione, and this inhibitory effect was further reinforced by the joint action of noradrenaline in the celiac ganglion and LH in the ovary. The levels of catecholamines in the ovarian compartment showed differences among the experiments; of significance, the joint treatment of noradrenaline in the celiac ganglion and LH in the ovary resulted in a remarkable increase in the ovarian levels of noradrenaline and adrenaline when compared to the effect achieved by either one of the compounds added alone.

**Conclusion:**

Our results demonstrate that the noradrenergic stimulation of the celiac ganglion reinforces the LH-induced inhibition of androstenedione production by the ovary of late pregnant rats, and that this effect is associated with marked changes in the release of catecholamines in the ovary.

## Background

The ovary is innervated by the ovarian plexus nerve and the superior ovarian nerve [1], the latter being the most related to ovarian steroidogenesis [2]. Most of the fibers of the superior ovarian nerve come from the postganglionic sympathetic neurons of the celiac ganglion [3]. They directly innervate the ovarian theca and secondary interstitial cells and exert an indirect action on the luteal cells [4]. Neurotransmitters of peptidergic and catecholaminergic nature have been detected in the ovarian nervous terminals of the superior ovarian nerve [5], some of which are also present in the celiac ganglion [6]. Moreover, the presence of intrinsic ovarian neurons in several mammalian species, including rats, has been demonstrated [7-9]. Among the neurotransmitters expressed in these cells are nitric oxide (NO), neuropeptide Y (NPY) and catecholamines [10]. The celiac ganglion is part of the sympathetic prevertebral chain possessing a great variety of specific receptors and neurotransmitters such as catecholamines, neuropeptides, and nitric oxide [3,11,12], and constitutes a modulation center in the pathway of the afferent and efferent fibers between the central nervous system and the ovary [2]. The main preganglion neurotransmitter of the celiac ganglion is acetylcholine [13-15], yet the celiac ganglion-mesenteric complex also contain α and β adrenergic receptors and is innervated by fibers of adrenergic nature that come from other preaortic ganglia [6,16]. The presence of such receptors in the celiac ganglion has been demonstrated physiologically in adult [17,18] and prepubertal rats [19].

Our laboratory has previously demonstrated that modifications in the adrenergic activity of the celiac ganglion results in an altered capacity of the ovary of pregnant rats to produce progesterone [17], suggesting that the celiac ganglion-superior ovarian nerve-ovarian axis provides a direct link between the autonomic nervous system and the physiology of pregnancy (reviewed in [2]). More recently, we have also shown that modifications in the cholinergic input at the celiac ganglion also led, via the superior ovarian nerve, to modifications in ovarian steroidogenesis [20]. Adding a level of complexity to the understanding of the neuroendocrine interactions between the celiac ganglion and the physiology of the ovary we showed that modifications in the hormonal environment that surrounds the celiac ganglion affects its behavior, ultimately affecting ovarian steroidogenesis [2,21].

Androstenedione is a major luteotropic hormone for the pregnant rat ovary. The involvement of androstenedione is widely accepted in the maintenance of luteal function upon its intraluteal conversion to estradiol [22]. Moreover, it has been demonstrated in different experimental schemes that androstenedione has also a direct luteotropic effect stimulating luteal progesterone production – an effect not mediated by its previous conversion to estradiol – [23-25], and interfering with the programmed cell death process that accompanies the regression of the corpus luteum [25]. More recently, our laboratory demonstrated that androstenedione can also indirectly exert a luteotropic effect by regulating the activity of the celiac ganglion [21].

In pregnant rats LH has a peculiar dual effect controlling ovarian function. During the first half of pregnancy LH is essential, together with prolactin, to sustain the corpus luteum [22], whereas its increase in late pregnancy is detrimental for luteal function, promoting the regression of the corpora lutea that accompanies parturition [26-28]. Considering the relevant role of androstenedione in rat pregnancy and the fine modulatory control exerted by the peripheral neural system throughout the celiac ganglion on pregnant rat ovarian steroidogenesis, we set up to study whether the celiac ganglion modulates, via the superior ovarian nerve, the anti-steroidogenic effect of LH in late pregnant rats as assessed by measuring the production of androstenedione by the ovary.

## Materials and methods

### Animals

Pregnant (day 0 = sperm positive) Holtzman rats were used in the studies. They were housed under controlled conditions of light (lights on 07:00 – 19:00 h) and temperature (24 ± 2°C) with free access to standard rat chow and water. Animals were handled in accordance with to the procedures approved in the UFAW Handbook on the Care and Management of Laboratory Animals [29]. The experimental protocol was approved by the University of San Luis Animal Care and Use Committee (ordinance CD 006/02).

### Drugs

The following chemicals were purchased from Sigma Chemical Co. (St. Louis, MO, USA): L-D-noradrenaline hydrochloride (NA), dextrose, ascorbic acid, bovine serum albumin fraction V (BSA), and luteinizing hormone (LH). 1, 2, 6, 7-[^3^H] androst-4-ene-3, 17-dione (115.0 Ci/mmol) was provided by New England Nuclear Products (Boston, MA, USA). Other reagents were of analytical grade.

### Experimental procedure

**Figure 1:**
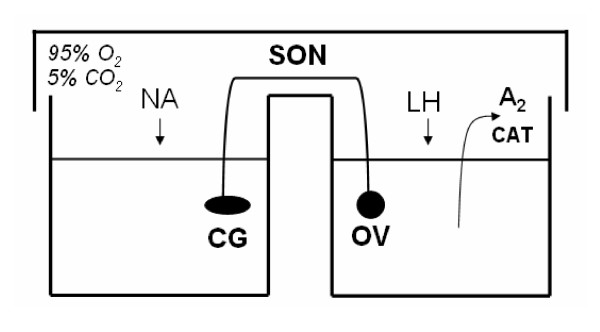
Schematic representation of the celiac ganglion-superior ovarian nerve-ovary ex vivo incubation system. NA, noradrenaline; CG, celiac ganglion; SON, superior ovarian nerve; OV, ovary; CAT, catecholamines; A_2_, androstenedione.

Groups of six animals on days 15, 19, 20 and 21 of pregnancy were used. They were anaesthetized and the celiac ganglion-superior ovarian nerve-ovary system was removed by dissection avoiding contact between the surgical instruments and the nerve fibers, in order to prevent spontaneous depolarization of the nerves. The piece of tissue consisted of the left ovary, the fibers that constitute the superior ovarian nerve inserted in the suspensory ligament, and the celiac ganglion accompanied by some small surrounding ganglia. The total surgical procedure was completed in 1–2 min. In order to verify the existence of the ganglion, routine histological techniques were followed throughout the characterization of the system (results not shown). Once the celiac ganglion-superior ovarian nerve-ovary system was removed, it was cleaned with incubation solution and placed immediately in a cuvette with two compartments. Each compartment contained 2 ml of buffer Krebs Ringer-bicarbonate pH 7.4, with the addition of 0.1 mg/ml glucose and 0.1 mg/ml albumin. The celiac ganglion was placed in one compartment and the ovary in the other, connected by the superior ovarian nerve, which had to be kept moist with the work solution (Figure 1). The system was stabilized by preincubation in a metabolic bath at 37°C for 30 min under a saturated O_2_/CO_2 _(95:5%) atmosphere. The end of the pre-incubation period was considered as incubation time 0. At this time, the buffer was changed in both compartments and 0.1 mg/ml ascorbic acid was added as an antioxidant to the ganglion compartment (**Control group**). For the experimental groups at incubation time = 0: 1) noradrenaline (10^-6 ^M) was added in the ganglion compartment (**NA group**); 2) on day 15 of pregnancy, LH (50 ng/ml) was added in the ovarian compartment (**Control-LH**); 3) on day 15 of pregnancy, LH (50 ng/ml) was added in the ovarian compartment and noradrenaline (10^-6 ^M) was added in the ganglion compartment (**NA-LH group)**. Periodic extractions were made from the ovarian compartment at 30, 60, 120 and 180 min for determination of androstenedione and catecholamines (180 min) released in the incubation media.

### Hormone determination

The samples of liquid from the ovarian compartment were stored at -20°C until hormone assay. Androstenedione was measured by radioimmunoassay (RIA); the variability and cross-reaction of this RIA have been previously reported [25]. The assay sensitivity was less than 10 pg/ml androstenedione and the intra-assay coefficient of variation was less than 10.0%. The results were expressed as pg of androstenedione per mg of ovarian tissue (A_2 _pg/mg ovary) against time of incubation. The corresponding corrections were made in all cases, taking into consideration the volume extracted in each tested period.

### Determination of catecholamines by HPLC

The catecholamines measured were dihydroxyphenylalanine (DOPA), dopamine (DA), noradrenaline (NA) and adrenaline (A). The catechols in 20 μl aliquots of liquid from the ovarian cuvette (180 min) were partially purified by batch alumina extraction, separated by reverse-phase high-pressure liquid chromatography using a 4.6 mm × 250 mm Zorbax R_x_C_18 _column (Du Pont, USA) and quantified by current produced upon exposure of the column effluent to oxidizing and then reducing potentials in series using a triple-electrode system (Coulochem II, ESA, Bedford, MA) [30]. Recovery through the alumina extraction step averaged 70–80% for catecholamines and 45–55% for DOPA. Catecholamine concentrations, in each sample, were corrected for recovery of an internal standard dihydroxybenzylamine. Levels of DOPA were further corrected for differences in recovery of the internal standard and this catechol in a mixture of external standards. The results were expressed as pg of catecholamine per mg of ovarian tissue/180 min incubation (Catechol pg/mg ovary/180 min incubation).

### Statistical analysis

For multiple comparisons made along the time of incubation, repeated measures analysis of variance followed by Tukey's test was used. Instead, for multiple comparisons not involving repeated measures, one-way analysis of variance followed by Tukey's test was utilized. A difference was considered to be statistically significant at P < 0.05 [31].

## Results

### Effect of the addition of noradrenaline in the ganglion compartment on the levels of androstenedione accumulated in the ovarian compartment of celiac ganglion-superior ovarian nerve-ovary preparations obtained from animals sacrificed on days 19, 20, and 21 of pregnancy

**Figure 2:**
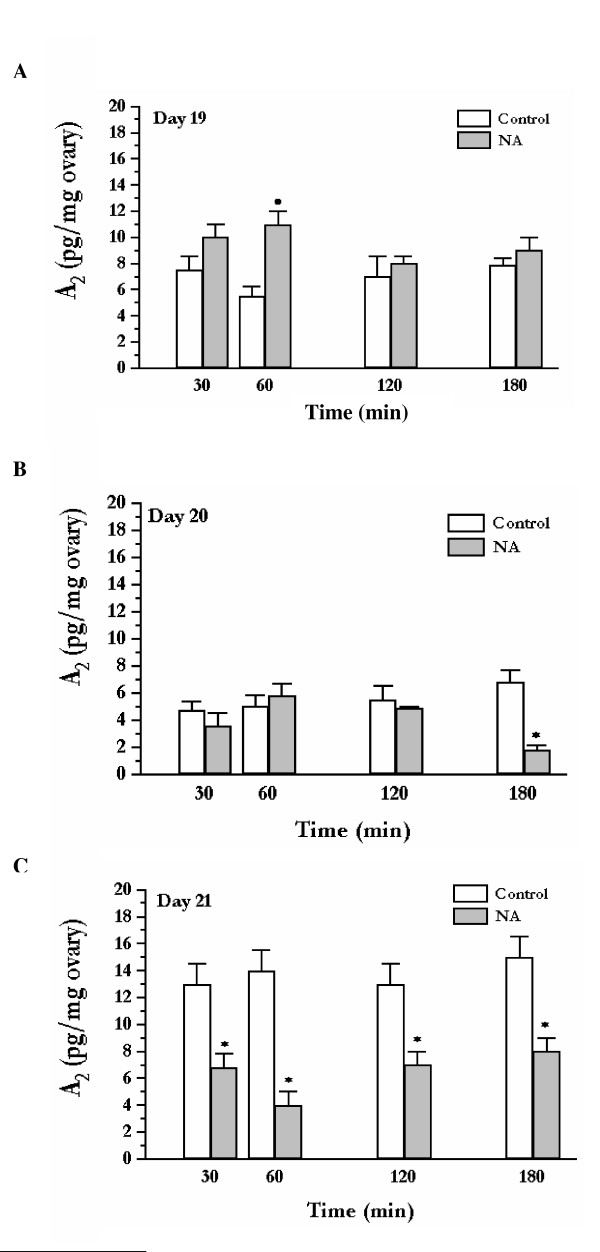
Effect of noradrenaline (NA) in the ganglion compartment on the release of ovarian androstenedione (A_2_), using the celiac ganglion-superior ovarian nerve-ovary system obtained from pregnant rats on days 19 (A), 20 (B) and 21 (C). The systems were incubated in Krebs-Ringer solution, at 37°C in an atmosphere of 95% O_2_-5% CO_2 _for 180 min. Ascorbic acid (0.1 mg/ml in Krebs-Ringer) without (Control) and with 10^-6 ^M NA was added to the ganglionic compartment (NA groups). Results are expressed as mean ± S.E.M. of six animals per group. Repeated measures analysis of variance followed by Tukey's test was used; * p < 0.01 and ^•^p < 0.05.

The levels of androstenedione accumulated in the ovarian compartment under basal conditions in the present studies were similar to those obtained previously in our laboratory [20]. The addition of the adrenergic agonist noradrenaline (NA; 10^-6 ^M) to the celiac ganglion led to significant changes in the amount of androstenedione accumulated in the ovarian compartment (Figure 2). In tissue preparations obtained from day 19 pregnant animals, the addition of noradrenaline to the ganglion compartment led to an increase in androstenedione accumulated in the ovarian compartment at 60 min incubation (^•^p < 0.05) (Figure 2A). In preparations obtained from day 20 pregnant animals, noradrenaline induced a decrease in the amount of androstenedione accumulated in the ovarian compartment at 180 min of incubation (* p < 0.01) (Figure 2B). Finally, on day 21 of pregnancy preparations, the presence of noradrenaline in the ganglion compartment caused a decrease in the levels of androstenedione accumulated in the incubation media when compared to controls along all times studied (* p < 0.01) (Figure 2C).

### Effect of the addition of noradrenaline in the ganglion compartment and LH in the ovarian compartment on the levels of androstenedione accumulated in the ovarian compartment of celiac ganglion-superior ovarian nerve-ovary preparations obtained on day 15 of pregnancy

**Figure 3:**
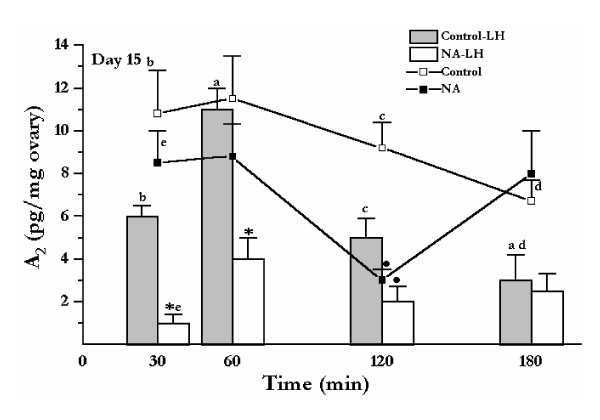
Effect of noradrenaline (NA) in the celiac ganglion and LH in the ovary on the production of ovarian androstenedione (A_2_), using the celiac ganglion-SON-ovary system obtained from rats on day 15 of pregnancy. The systems were incubated in Krebs-Ringer solution, at 37°C in an atmosphere of 95% O_2_-5% CO_2 _for 180 min. LH (50 ng/ml) was added to the ovarian compartment and ascorbic acid (0.1 mg/ml in Krebs-Ringer) without (Control-LH group) and with NA (10^-6 ^M) added to the ganglion compartment (NA-LH group). Control group was in absence of NA in the ganglion compartment and LH in the ovarian compartment. In the NA group, 10^-6 ^M NA was added to the ganglionic compartment. Results are expressed as mean ± S.E.M. of six animals per group. Repeated measures analysis of variance followed by Tukey's test was used. The same font denote differences of statistical significance; ^a^p < 0.05 between 60 and 180 min of Control-LH group; ^b,c ^and ^d ^p < 0.05 in Control-LH group compared with Control group; ^e^p < 0.01 NA-LH group compared with NA group.; *p < 0.01 and ^• ^p < 0.05 in NA-LH group compared with Control-LH group; ^• ^p < 0.05 in NA group compared with Control group.

When noradrenaline was added to the celiac ganglion compartment of the celiac ganglion-superior ovarian nerve-ovarian preparation obtained from day 15 pregnant rats, the concentration of androstenedione accumulated in the ovarian compartment decreased significantly at 120 min of incubation when compared to controls (^• ^p < 0.05). In Control-LH group, the presence of LH (50 ng/ml) in the ovarian compartment caused a significant decrease in the amount of androstenedione accumulated in the culture media at 180 min respect to 60 min (^a^p < 0.05). The presence of LH (50 ng/ml; Control-LH) in the ovarian compartment caused a significant decrease in the amount of androstenedione accumulated in the culture media at 30, 120 and 180 min when compared to the levels of androstenedione measured in the incubation media of ovarian preparations not exposed to LH (^b,c ^and ^d ^p < 0.05 vs. time-matched controls). The combined presence of noradrenaline (10^-6 ^M) in the ganglion compartment and LH in the ovarian compartment decreased, up to 120 min, the levels of androstenedione accumulated in the media when compared with incubation media of ovaries stimulated with LH but without the presence of noradrenaline in the ganglion compartment (NA-LH vs. Control-LH) (30 and 60 min: * p < 0.01; 120 min: ^• ^p < 0.05). NA-LH group decreased the levels of androstenedione at 30 min compared with the NA group ^e ^p < 0.01. (Figure 3).

### Effect of the addition of noradrenaline in the ganglion compartment and LH in the ovarian compartment on the levels of catecholaminergic neurotransmitters released in the ovarian compartment of celiac ganglion-superior ovarian nerve-ovary preparations obtained on day 15 of pregnancy

**Figure 4:**
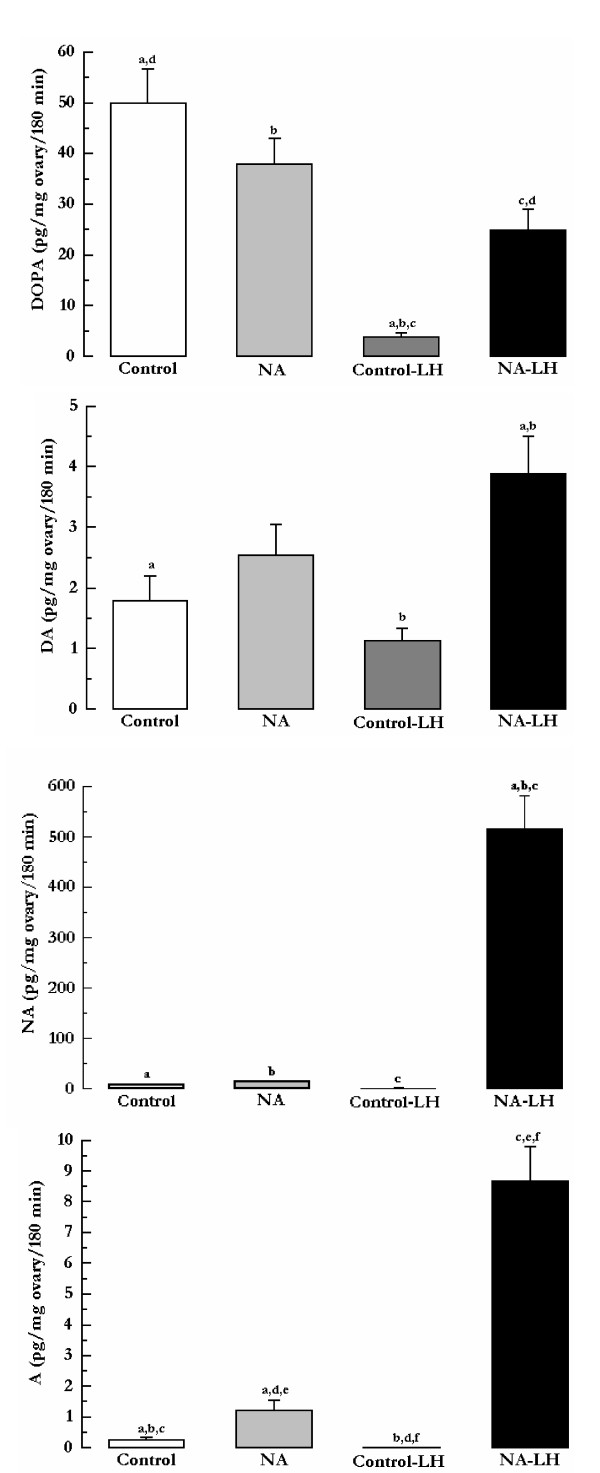
Levels of catecholamines in the incubation liquid of the ovarian compartment at 180 min using the celiac ganglion-superior ovarian nerve-ovary system obtained from rats on day 15 of pregnancy. The systems were incubated in Krebs-Ringer solution, at 37°C in an atmosphere of 95% O_2_-5% CO_2 _for 180 min. LH (50 ng/ml) was added to the ovarian compartment and ascorbic acid (0.1 mg/ml in Krebs-Ringer) without (Control-LH group) and with NA (10^-6 ^M) added to the ganglion compartment (NA-LH group). Control group was in absence of NA in the ganglion compartment and LH in the ovarian compartment. NA group was in absence of LH in the ovarian compartment. Dihydroxyphenylalanine (DOPA), dopamine (DA), noradrenaline (NA), adrenaline (A). Results are expressed as mean ± S.E.M. of six animals per group. One-way analysis of variance followed by Tukey's test was used. Control, NA, Control-LH and NA-LH groups were compared. The same fonts denote differences of statistical significance.

The celiac ganglion-superior ovarian nerve-ovary system obtained from 15-day pregnant rats was used to investigate the presence of various catecholaminergic neurotransmitters in the ovarian incubation liquid. When analyzing the levels of the neurotransmitters accumulated up to 180 min of the system incubation, it was observed that these levels were modified among the experimental paradigms. In relation to dihydroxyphenylalanine (DOPA) (Figure 4A), the highest levels were those observed in the Control group. The presence of LH in the ovarian compartment significantly decreased the concentration of DOPA when compared to the control (Control-LH vs. Control; ^a ^p < 0.01) and to the group receiving noradrenaline in the celiac ganglion compartment (NA group) (^b ^p < 0.05). However, the simultaneous presence of noradrenaline in the ganglion compartment significantly prevented the decrease in DOPA accumulation induced by LH (^c ^p < 0.05), but without reaching the control levels (^d ^p < 0.05). In Figure 4B, it can be observed that the levels of dopamine (DA) in the ovarian compartment increased significantly upon the combined addition of noradrenaline in the ganglion compartment and LH in the ovarian compartment (NA-LH group) when compared to Control (^a ^p < 0.05) and Control-LH (^b ^p < 0.05) groups. The only addition of noradrenaline in the celiac ganglion or LH in the ovarian compartment did not show significant differences in relation to the control levels. Figure 4C shows that the levels of noradrenaline are very low in the Control group, in the group having noradrenaline in the ganglion compartment (NA), and in the group receiving LH in the ovarian compartment (Control-LH). However, there was a remarkable increase in the amount of noradrenaline accumulated in the media of the ovarian compartment with the simultaneous presence of noradrenaline in the ganglion compartment and LH in the ovarian compartment (NA-LH) (^a,b ^and ^c ^p < 0.01). Figure 4D shows that adrenaline (A) was also present in the ovarian incubation liquid, although at very low levels. These levels were further decreased by the presence of LH in the ovarian compartment (^b ^p < 0.05). The addition of noradrenaline in the ganglion compartment caused a slight increase in the amount of adrenaline accumulated in the ovarian compartment in relation to the Control and the Control-LH groups (^a ^and ^d ^p < 0.05), whereas the concurrent presence of LH in the ovarian compartment and noradrenaline in the ganglion compartment led to a noteworthy increase in the amount of adrenaline accumulated in the ovarian compartment in relation the three previous situations (^c,e ^and ^f ^p < 0.01).

## Discussion

The celiac ganglion-superior ovarian nerve-ovary system utilized in this study provides an adequate ex vivo model with good resemblance to the in vivo conditions, and allows discerning endocrine-neural interactions about the regulation of the physiology of the ovary. In the present study, using this experimental approach, we present evidence demonstrating that the capacity of the ovary of late pregnant rats to produce androstenedione is modulated by neural inputs coming from the celiac ganglion via the superior ovarian nerve. This study also confirms that there is a functional inter-relationship between the adrenergic inputs on the celiac ganglion and the endocrine action of LH in the ovary and that the response of the ovary to neural stimulation varies throughout pregnancy. Furthermore, these results contribute to the understanding of the role of the peripheral nervous system in ovarian physiology and can add to the understanding of certain pathologic states of reproduction that cannot be solely explained by hormonal causes, such as polycystic ovary syndrome, whose development is profoundly influenced by the sympathetic nervous system [32].

In celiac ganglion-superior ovarian nerve-ovary preparations obtained on days 15, 19, and 20 of pregnancy noradrenergic stimulation of the celiac ganglion did not largely modify the capacity of the ovary to produce androstenedione; however, there was a marked inhibition in the capacity to accumulate androstenedione in the ovaries of preparations taken from day 21 pregnant rats that had been subjected to noradrenergic stimulation of the celiac ganglion. It is possible that ovaries of day 21 pregnant rats respond strongly to neural inputs because androstenedione most probably originates in the interstitial cells and the theca cells of growing preovulatory follicles found in the ovary of rats at term [22] and that are directly innervated by terminals from the superior ovarian nerve [4]. The inhibitory effect of the noradrenergic stimulation of the celiac ganglion on the androstenedione producing capacity of late pregnant ovaries was further visualized by the enhancement of the anti-steroidogenic effect of LH. We have previously shown that adding LH to the ovarian compartment of celiac ganglion-superior ovarian nerve-ovary preparations obtained from day 15 pregnant rats did not affect progesterone production, yet when combined with noradrenergic stimulation of the celiac ganglion led to a decrease in the ovarian progesterone producing capacity [17]. Overall results from the present work and previous work in our laboratory [17] demonstrate that the anti-steroidogenic effect of LH in late pregnant rat ovaries is modulated by the activity of the celiac ganglion. Whereas LH alone may be sufficient to impair the capacity of the ovary to produce androstenedione, it may need the contribution of neural inputs coming from the celiac ganglion via the superior ovarian nerve to also impair progesterone production.

The luteotropic effect of androstenedione in pregnant rats has been widely demonstrated in different experimental schemes [21,23,25]. Androstenedione appears to be a potent tropic hormone that assists corpus luteum function particularly at the end of pregnancy when the gland approaches its regression. This is supported by the fact that when the ovary is populated with corpora lutea that are approaching the time of regression (day 21 of pregnancy), they become more responsive to androstenedione in terms of progesterone production in vivo and in vitro [24]. Our current data show that ovaries taken at end of pregnancy (day 21) are capable of producing more androstenedione in vitro than ovaries obtained on previous days of pregnancy. Consequently, in a physiological setting, it is possible that the ovarian production of androstenedione needs to be limited in order to ensure the regression of the corpus luteum. Neural inputs coming from the celiac ganglion may be in charge of fine-tuning, together with LH, the production of androstenedione at the end of pregnancy.

In the current study we also provide evidence that four metabolically related catecholamines (dihydroxyphenylalanine, dopamine, noradrenaline and adrenaline) are detected in the ovarian incubation compartment of the celiac ganglion-superior ovarian nerve-ovary tissue preparation. The increase observed in the accumulation of noradrenaline and adrenaline within the ovarian compartment upon the joint adrenergic stimulation of the celiac ganglion and the endocrine ovarian effect of LH was particularly dramatic, suggesting that they may be involved in the anti-steroidogenic effect of LH at the end of pregnancy in rats. It is not possible from our experiments to know, however, the source or location of these amines. LH may locally induce the release of catecholamines either from: i) the nervous terminals coming from the peripheral innervation (pool of catecholamines synthesized in the celiac ganglion or in the superior ovarian nerve terminals) [1]; ii) the intrinsic neurons of the ovary (structures from a network of neurons developed as a ganglion and located in the meso-ovarium and hilium and neurons mostly isolated in the cortex and medulla) [10]; or iii) a combination of both.

It was noticeable that the anti-steroidogenic effect of LH on androstenedione production was associated with elevated adrenaline and noradrenaline in the ovarian compartment; this observation is controversial because of the fact that these two catecholamines have been usually reported as favoring steroidogenesis [2]. It is possible that any action of the catecholamines in the ovary in the presence of elevated concentrations of LH could be prevented by a desensitizing effect of the gonadotropin in the coupling of beta adrenergic receptors to adenylate cyclase [33]. We should also consider that probably other neurotransmitters not analyzed in this work, such as NO [34,35], GnRH [36,37] or neuropeptides such as NPY and substance P (SP) [38] play roles together with catecholamines in the neural control of the function of the ovary during pregnancy; consequently they may mediate the anti-steroidogenic effect of LH in the presence of elevated catecholamines.

There is ample evidence suggesting that the sympathetic nervous system is involved in pathologies associated to the reproductive system. Evidence has been accumulating supporting the participation of sympathetic nerves in the function of the ovary under normal and pathological conditions such as polycystic ovarian syndrome [32,39]. It is overall suggested that chronic increase in ovarian sympathetic nerve activity is related to changes in follicular development, producing an anovulatory ovary with cysts, but that the process can be reversed by attenuation of the sympathetic activity [40]. Polycystic ovarian syndrome is a common cause of infertility in women during their reproductive years and is associated with cystic ovaries and increased capacity to produce androgens. Whether a dysregulation in the activity of the celiac ganglion and the superior ovarian nerve is involved in the dysregulated androgen production observed in polycystic ovary syndrome remains an attractive subject for future investigations. The results of the present study demonstrating that adrenergic activation of the celiac ganglion impacts ovarian androgen production in rats, certainly stimulates such future investigations.

## Conclusion

Our results demonstrate that in the ovary of late pregnant rats the production of androstenedione is inhibited upon the joint action of noradrenaline in the celiac ganglion and LH in the ovary, and that this effect is associated with marked changes in the release of catecholamines in the ovary.

## Authors' contributions

MC carried out most of the experiments, performed the statistical analysis and has been involved in drafting and writing the manuscript. SMD carried out some of the experiments and contributed to the statistical analysis. ZS made substantial contributions to conception and design of the study and has been involved in revising the manuscript critically for important intellectual content. CMT has made substantial contributions to analysis and interpretation of data and has been involved in revising the manuscript critically for important intellectual content. AMR made substantial contributions to conception and design of the study and has been involved in drafting and writing the manuscript. The authors read and approved the final manuscript.
